# Colony size affects nestling immune function: a cross-fostering experiment in a colonial waterbird

**DOI:** 10.1007/s00442-019-04402-3

**Published:** 2019-04-19

**Authors:** Piotr Minias, Kamila Gach, Radosław Włodarczyk, Tomasz Janiszewski

**Affiliations:** Department of Biodiversity Studies and Bioeducation, Faculty of Biology and Environmental Protection, University of Łódź, Banacha 1/3, 90-237 Łódź, Poland

**Keywords:** Coloniality, Common tern, Cross-fostering, Immune response, Social stress

## Abstract

Elevated transmission rate of pathogens and parasites is considered one of the major costs of sociality in birds. However, greater risk of infection in colonial birds might be compensated by specific immune adaptations. Here, we predicted that nestlings raised in larger colonies should invest more in their immune function. To test this hypothesis, we manipulated colony size and conduced cross-fostering experiment in a colonial waterbird, the common tern *Sterna hirundo*. Establishment of different size colonies under uniform environmental conditions was induced by providing large and small patches of attractive nesting area for terns (floating rafts). Then, pairs of clutches were swapped between large and small tern colonies, and skin-swelling response to phytohaemagglutinin (PHA) was assessed for nestlings from experimental and control broods. Contrary to our expectations, we found a negative effect of foster colony size on nestling PHA response (nestlings raised in the larger colony had lower PHA response). In addition, nestling PHA response correlated negatively with heterophil/lymphocyte ratio used as a measure of physiological stress. This suggested that low PHA response of nestlings raised in the larger colony could be mediated by an elevated level of social stress. We suggest that depression of immune function via social stress may constitute a strong selective pressure against large colony size in the common tern, and possibly in other colonial species. We also recommend that this largely overlooked cost of sociality should be considered in the further studies on the evolution and ecology of avian coloniality.

**Electronic supplementary material:**

The online version of this article (10.1007/s00442-019-04402-3) contains supplementary material, which is available to authorized users.

## Introduction

Coloniality occurs in different groups of vertebrates including fish, reptiles, and mammals, but it is most widespread in birds, as approximately 13% of all avian species are classified as colonial breeders (Lack 1968). Despite extensive research on the evolution and function of coloniality (Brown and Brown 2001), the relative importance of ecological and environmental factors that produce inter- and intra-specific variation in colony size is still being debated (Brown 2016). Two of the most commonly evoked benefits of breeding in large aggregations include reduction in predatory pressure by means of communal vigilance and defense (Götmark and Andersson 1984; Wiklund and Andersson 1994), as well as enhancement of individual foraging efficiency by making observations on where and how other members of a colony locate food (Richner and Heeb 1995). On the other hand, living in large social groups carries the costs of elevated intra-specific competition and higher transmission rates of parasites and disease. In fact, elevated pathogen pressure is considered a universal hazard to all group-living animals (Côté and Poulin 1995) and it has been argued as one of the major costs of breeding sociality in birds (Tella 2002).

It might be expected that greater risk of infection from a greater diversity of pathogens in colonial species should be compensated by specific adaptations of their immune system. For example, it has been shown that highly social species of birds develop larger immune defense organs (bursa of Fabricius and spleen) than solitary species (Møller and Erritzøe 1996). In addition, the strength of T- and B-cell immune response positively correlated with degree of sociality in swallows and martins Hirundinidae (Møller et al. 2001). Much less is known on whether analogical immune adaptations occur intra-specifically in response to colony size. There is large body of evidence that horizontal transmission rate of pathogens increases with colony size (Brown and Brown 1986; O’Brien and Brown 2011), so individuals that settle in larger aggregations should invest more in immune response. Higher investment in pathogen resistance would be also expected in nestlings that are raised in larger colonies, but an extensive correlational study on the Magellanic penguin *Spheniscus magellanicus* provided contrary evidence. It has been shown that T-cell-mediated immune response of penguin nestlings negatively covaried with colony size (500–175,000 breeding pairs) and this pattern was attributed to high level of social stress (defined as physiological stress reaction stemming from social interactions or general social environment) or poor nutritional condition in large colonies (Tella et al. 2001), which was suggested to have a detrimental effect on individual ability to prevent and control infection (i.e., immunocompetence). Nevertheless, these results were obtained by comparing different size colonies from various geographical locations, which were likely to be subject to different environmental and ecological conditions. In fact, the effect of colony size per se on nestling immunocompetence might be difficult to disentangle from the background variation in such factors as location-specific parasitic pressure or food availability without a proper experimental design.

In this study, we used an experimental approach to assess the effect of colony size on nestling immune function in a colonial larid species, the common tern *Sterna hirundo*. First, we induced formation of different size colonies under uniform environmental conditions by providing large and small patches of attractive nesting area for common terns (floating rafts) at a site with no access to natural nesting habitat. Second, we performed a cross-fostering experiment to examine whether any possible effect of colony size on nestling immune function was due to rearing conditions (foster colony effect) or due to genetic factors or maternal (paternal) effects (colony-of-origin effect). We expected that nestling immune function may be related to the size of the foster colony, because chicks raised in larger aggregations should invest more in immune response due to elevated pathogen transmission rate. On the other hand, common tern nestlings raised in larger colonies were shown to suffer increased social stress and poor nutritional condition (Minias et al. 2015), which can have detrimental effect on immunological capability. Nestling immune function can also be associated with the original colony size, as adults that choose to breed in larger aggregations should have stronger innate immune resistance, which can be inherited or maternally transmitted to chicks. To test these hypotheses, we swapped pairs of clutches between large and small colonies of the common tern, and assessed skin-swelling response to phytohaemagglutinin (PHA) in nestlings from experimental and control broods. Despite complex immunological background of the PHA response (Owen and Clayton 2007; Vinkler et al. 2010), it is one of the most common immune indices used in ecology as a measure of acquired T-cell-mediated immune response (Kennedy and Nager 2006; Tella et al. 2008). Because immune function (as measured with PHA response) is known to depend on nutritional condition (Alonso-Alvarez and Tella 2001), we also tested whether between-colony variation in this trait was mediated by differences in growth rate, haematocrit, or plasma metabolite concentrations of common tern nestlings raised in the colonies of different size. Finally, to test whether between-colony variation in chick PHA response was mediated by different levels of social stress, we assessed nestling heterophil/lymphocyte (*H*/*L*) ratio, which is considered a reliable proxy of physiological stress in birds (Davis et al. 2008).

## Material and methods

### Study site and experimental colonies

Fieldwork was conducted in 2013–2014 at the Jeziorsko reservoir (51°40′N, 18°40′E), central Poland. Data were collected in four experimental colonies of common terns. To provoke establishment of different size colonies, we installed one large (ca. 40 m^2^) and three small (ca. 10 m^2^) artificial nesting rafts at the site with no natural nesting habitat for common terns. All rafts were square-shaped and enclosed with a 30 cm-high fence (3 mm mesh size), so that the chicks could not leave the platforms until fledging. Raft surface was about 0.5 m above the water level to avoid flooding of nests, and it was covered with sand and gravel. The rafts were installed in 2011 within protected area of Jeziorsko nature reserve to avoid human disturbance during the breeding season. All rafts were located in open water about 1 km from the shore, but relatively close to inundated riparian willow shrubs (50–100 m), which reduced wave impact. Localization of rafts was chosen to maximize distances between them (150–500 m), so that each colony functioned as an independent unit. Field observations seemed to support our expectation that interactions between terns nesting at different rafts were limited. Adults and juveniles from other rafts were observed to be chased away by locally breeding birds, and we obtained no evidence of adults residing on rafts others than the one with their own brood (based on resightings of birds marked with plastic rings). Breeding colonies were established in 2011 and colony size during the study period varied from 105–110 pairs at the large raft to 25–35 pairs at the small rafts. Nesting densities were similar at all rafts (2.5–3 nests/m^2^ during nesting peak in mid-June).

### General field procedures and cross-fostering

Starting from the beginning of May, all experimental colonies were visited every 3–5 days. The first clutches were laid on 05–10 May. During each visit, we marked all new clutches with indelible marker and recorded laying dates. We randomly selected 55 pairs of clutches (27 in 2013 and 28 in 2014) to be exchanged between the colonies of different size (110 individual clutches in total). Clutches were paired according to the number of eggs (two and three-egg clutches included) and laying dates (± 2 days). The entire procedure of clutch exchange between two colonies was always completed within one hour and for the time of clutch transportation artificial eggs were deposited in experimental nests to avoid clutch desertion. The same number of clutches was selected as controls (55 clutches in the large colony and 55 clutches in the small colonies). Control clutches could not be exchanged within the colonies due to limited number of nests (especially in small colonies), but they were handled to account for potential effects of disturbance and put back to the nest of origin. During peak hatching period, the colonies were visited each 2–3 days to assign hatchlings to natal nests before they started to be mobile. All hatchlings were individually marked with numbered rings and their accurate hatching date was established based on morphological measurements fitted into the growth curve developed for known-age chicks (see details in Minias et al. 2015).

### PHA response measurements

Nestling immune function was measured as an inducible cutaneous response to the mitogen, phytohaemagglutinin (PHA). PHA challenge produces a prominent perivascular accumulation of T-lymphocytes followed by macrophage infiltration and it has been routinely used to quantify vertebrate T-cell-mediated immune response in various ecological contexts (Martin et al. 2006). Cross-fostered and control nestlings were injected subcutaneously in the left wing web with 0.2 mg PHA (Sigma-Aldrich Co., St. Louis, MO, USA) dissolved in 0.05 ml of physiological saline solution (phosphate-buffered saline; Sigma-Aldrich Co.). Nestling age varied between 10 and 24 days at the moment of injection. Thickness of wing web was measured with a digital pressure-sensitive micrometre (Mitutoyo, Kawasaki, Japan) before and 24 h after the injection. Each time, wing web thickness was measured three times. Repeatability of measurements (intra-class coefficients) was high (0.95 and 0.96 for pre- and post-injection measurements), as calculated using *irr* package (Gamer et al. 2012) developed for R statistical environment (R Development Core Team 2013). Following recommendations by Smits et al. (1999), we did not inject the second wing with a physiological saline solution as the experimental control. Differences in the average wing web thickness prior to and 24 h after injection (henceforth referred to as the PHA response) was used as a measure of nestling immune function. Due to relatively high clutch and brood losses, nestlings from only 42.7% of nests originally selected for the experiment were subjected to PHA challenge. The rate of brood failure was slightly lower in experimental than control broods (50.9% vs. 63.7%), but the difference was not statistically significant (*G* = 1.83, *df* = 1, *P* = 0.18). In total, PHA response was measured in 81 chicks from 54 broods exchanged between colonies of different size and 61 chicks from 40 control broods.

### Growth rate, condition, and physiological stress

To assess growth rate, we collected repeated measurements of wing length and body mass of common tern nestlings. Chicks were usually measured every 4–6 days [mean interval of 4.77 ± 0.05 (SE) days] from hatching until fledging (ca. 30 day of life). For 113 PHA-challenged chicks, we collected between five and nine [on average 6.04 ± 0.09 (SE)] measurements of wing length and body mass per individual. These measurements were fitted into logistic growth curves of the form *y* = *A*/[1 + *B* × exp(−KT)], where *y* is the body measurement at age *T*, *A* is an asymptotic value, *B* is a constant of integration, and *K* is the growth rate constant (Richner 1989). All curves well fitted to the data (all *R*^2^ > 0.90 for body mass and all *R*^2^ > 0.95 for wing length). Parameter *K* was used as an indicator of average chick growth.

Condition of nestlings at the time of PHA challenge was measured with haematocrit (the relative volume of red blood cells compared with total blood volume) and plasma concentrations of basic metabolites (total protein, albumin, triglycerides, and glucose). Although reliability of haematocrit as a condition index in wild birds remains under discussion (Fair et al. 2007), several studies have shown that haematocrit values decrease in response to nutritional stress (Piersma et al. 2000) and unpredictable feeding schedules (Acquarone et al. 2002; Cucco et al. 2002). In addition, plasma concentrations of basic metabolites have been reported to correlate with food intake, diet quality, mass gain, and fat reserves in a large spectrum of wild bird species (e.g. Totzke et al. 1999; Lyons et al. 2008). For the purpose of measurements, ca. 40 μL of blood was collected from the ulnar vein (right wing) of nestlings (*n* = 132) just prior to PHA injection. Blood was collected into heparinized capillary tubes, which were kept in portable refrigerators at 4 °C until they reached laboratory. Within 6 h from blood collection, all tubes were centrifuged at 6000 rpm for 5 min and haematocrit values were determined directly using a microhematocrit reader. Plasma was kept at − 20 °C until analysis. All plasma metabolite concentrations were analyzed with BTS-330 spectrophotometer (BioSystems Reagents & Instruments, Barcelona, Spain) using commercial kits of the same manufacturer (BioSystems Reagents & Instruments). All analyses were conducted according to the manufacturer’s protocol using the following methods: total protein (biuret reaction), albumin (bromocresol green), triglycerides (glycerol phosphate oxidase/peroxidase), and glucose (glucose oxidase/peroxidase). Absorbance of each sample was measured in a flow cuvette against a blank reagent. Since the quantity of plasma collected from each nestling was often not sufficient to measure concentrations of all four metabolites, sample sizes for each plasma parameter differed (sample size range 78–90 individuals).

For 123 PHA-challenged nestlings, blood smears of one cell layer were made on slides. Blood smears were air-dried and stored in darkness until analysis. All smears were stained using the May-Grünewald-Giemsa method and scanned at 1000× magnification under a light microscope. Leukocyte profiles were assessed as a relative proportion of five cell types (heterophils, lymphocytes, eosinophils, basophils, and monocytes) in a random sample of 100 white blood cells. To reduce variability, all blood smears were assessed by one of the authors (RW). Leukocyte profiles were used to calculate heterophil-to-lymphocyte (*H*/*L*) ratio, which is a general measure of physiological stress in birds and other vertebrates (Davis et al. 2008).

### Molecular sexing

For all PHA-injected nestlings (*n* = 142), we also collected 10 μL of blood from the ulnar vein (right wing) into Eppendorf tubes with 96% ethanol. Samples for molecular sexing were collected together with the other blood samples (for condition measurements) immediately before PHA challenge. Samples were stored at − 4 °C until analysis. DNA was extracted using a Genomic DNA Purification Kit (Thermo Fisher Scientific, Waltham, MA, USA) according to kit protocol. Molecular sexing was based on the sex-linked chromo-helicase-DNA-binding (CHD) gene, which was amplified using the primer pair 2550F and 2718R (Fridolfsson and Ellegren 1999). Products were separated on 2% agarose gel; males and females were identified with one and two bands, respectively.

### Statistical analyses

Nestling PHA response under the cross-fostering experiment was analyzed using the general linear mixed model, with the sizes of original and foster colonies (small vs. large) entered as fixed factors. Data from all three small colonies were treated jointly, as we found no differences in PHA response (*P* = 0.61), most condition indices (all *P* > 0.15, expect for haematocrit: *P* = 0.027), and leukocyte counts (all *P* > 0.30) between control broods from these colonies. The effects of age, sex, and year were included as fixed factors in the model, while hatching date was entered as a covariate. We also tested for the interactions of origin and foster colony size with age, sex, and hatching date. Since data from siblings were non-independent, we entered brood identity as a random factor to avoid pseudoreplication. To obtain more parsimonious reduced model, we removed non-significant (*P > *0.05) predictors from the initial full model, except for predictors included in significant interaction terms. To assess relationships of nestling PHA response with growth rate, condition indices (haematocrit and plasma metabolite concentrations), and leukocyte counts, we used partial correlation while controlling for the effects of age, sex, year, and hatching date. Distribution of all measured traits, except for haematocrit and plasma triglyceride concentration, was reasonably close to normal (absolute skewness and kurtosis < 1.5). To improve normality, haematocrit and plasma triglyceride concentration values were square- and log-transformed, respectively. Statistical analyses were performed in JMP 10 (SAS Institute Inc., Cary, NC, USA) and Statistica 10 (StatSoft, Tulsa, OK, USA). All values are reported as means ± SE.

## Results

**Fig. 1:**
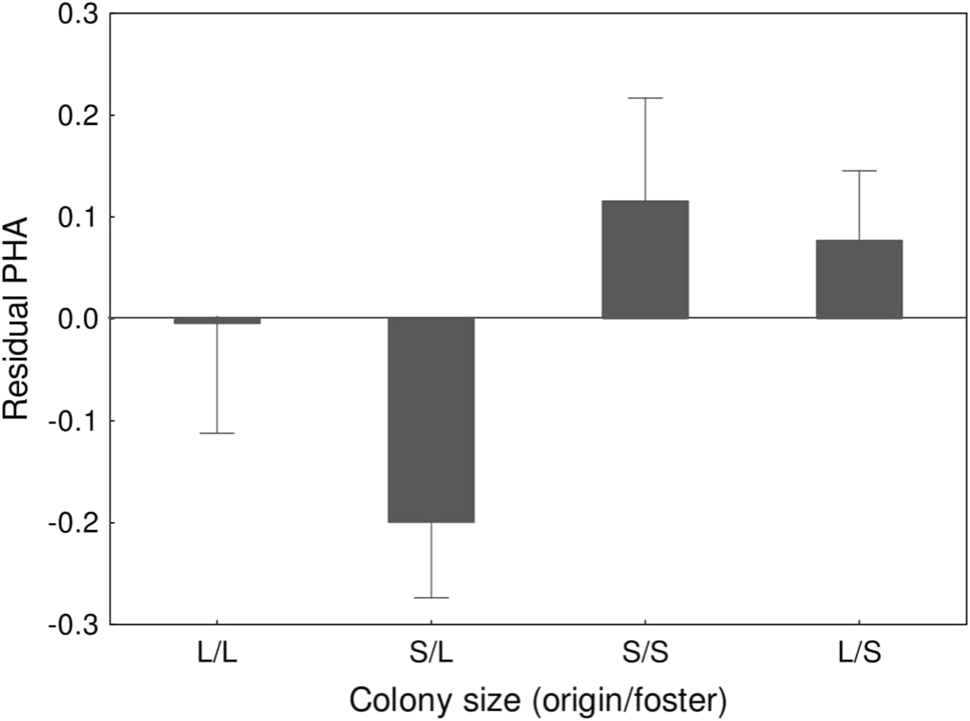
Residual PHA response in cross-fostered (*S*/*L* and *L*/*S*) and control (*L*/*L* and *S*/*S*) common tern broods. The effect of the colony of origin was not significant. Residual values were extracted from the reduced general linear mixed model; means ± SE are presented. *S* small colony, *L* large colony

We found no effect of original colony size on the nestling skin-swelling response to PHA (Table 1). In contrast, both full and reduced models indicated a significant effect of foster colony size on nestling immune function (Table 1; Fig. 1), as offspring raised in smaller colonies showed stronger PHA response (1.62 ± 0.05 mm vs. 1.44 ± 0.05 mm for small and large foster colonies, respectively). We also found a significant sex-related variation in nestling PHA response, male offspring showing stronger response than female offspring (1.64 ± 0.06 mm vs. 1.45 ± 0.04 mm for male and female offspring, respectively). No effect of hatching date was found on nestling PHA response (Table 1). There was a significant interaction between the effects of foster colony size and nestling age in the full model (*P* = 0.024; Table 1), suggesting that the effect (regression slope) of age on PHA response was lower in large versus small colonies (*β* = − 0.018 ± 0.009). However, the effect of the interaction lost significance in the reduced model (*P* = 0.077; Table 1) and we found no significant effect of age on nestling PHA response while analyzing small and large foster colonies separately (small foster colony: *β* = 0.022 ± 0.014, *P* = 0.13; large foster colony: *β* = − 0.014 ± 0.015, *P* = 0.33). All other tested interactions were non-significant and excluded from the final model (Table 1).

**Table 1:** Effects of original and foster colony size on PHA response of common tern nestlings

Predictor	*t*	*P*
Full model		
Intercept	0.13	0.90
Colony size (origin)	0.32	0.75
** Colony size (foster)**	**2.68**	**0.009**
** Sex**	**2.32**	**0.022**
Age	1.53	0.13
Hatching date (HD)	0.65	0.51
Year	1.59	0.12
Colony size (origin) × sex	0.01	0.99
Colony size (foster) × sex	0.90	0.37
Colony size (origin) × age	1.38	0.17
** Colony size (foster)** × **age**	**2.31**	**0.024**
Colony size (origin) × HD	0.60	0.55
Colony size (foster) x HD	1.41	0.16
Reduced model		
**Intercept**	**8.61**	** < 0.001**
**Colony size (foster)**	**2.22**	**0.029**
**Sex**	**2.46**	**0.015**
Age	0.41	0.68
Colony size (foster) × age	1.79	0.077

Significant predictors (*P* < 0.05) are marked in bold

**Fig. 2:**
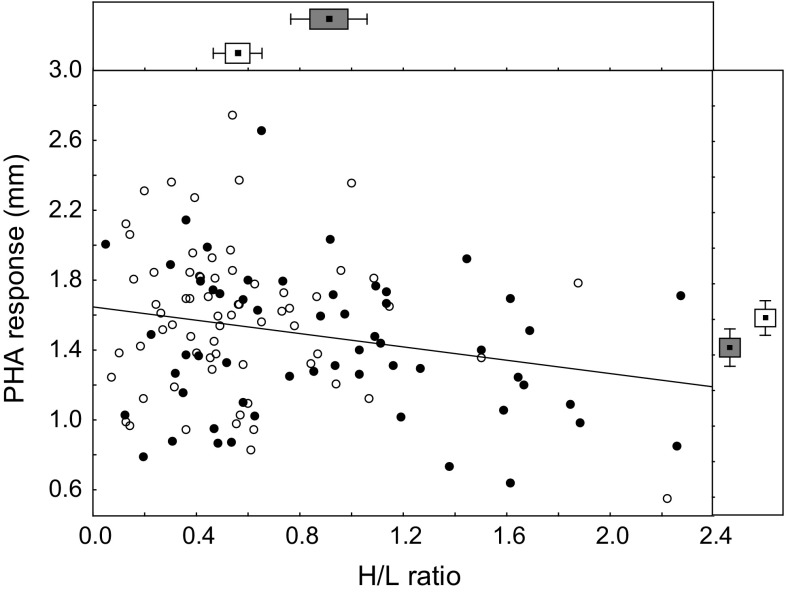
Relationship between PHA response and *H*/*L* ratio of common tern nestlings from large (black dots) and small (white dots) foster colonies. Summary statistics for PHA response and *H*/*L* ratio of common tern nestlings from large (grey box) and small (white box) foster colonies are shown at the right side and top panel, respectively (point—mean, box—SE, whiskers—95% confidence intervals).

After controlling for the effects of sex, age, year, and hatching date, we found a significant partial correlation between PHA response and *H*/*L* ratio of tern nestlings (Table 2). The correlation was negative, indicating that nestlings with higher *H*/*L* ratio showed weaker PHA response (Fig. 2). However, the relationship lost significance after controlling for an additional effect of foster colony size (*r* = − 0.16, *P* = 0.091). In fact, there were significant differences in nestling *H*/*L* ratio between foster colonies of different size (*P* = 0.001), as chicks raised in smaller colonies had lower *H*/*L* ratio (0.56 ± 0.05 vs. 0.91 ± 0.07 for small and large foster colonies, respectively; Fig. 2). We found no significant correlations between PHA response and absolute counts of heterophils and lymphocytes (Table 2). In addition, PHA response did not correlate with growth rate, hematocrit, and plasma metabolite concentrations of common tern nestlings (Table 2).

**Table 2:** Partial correlations (controlling for sex, age, year, and hatching date) of PHA response with growth rate and blood parameters of common tern nestlings

Trait	*r*	*P*
Body mass growth rate	0.05	0.64
Wing length growth rate	0.08	0.38
Haematocrit	− 0.03	0.76
Total plasma protein	− 0.07	0.55
Plasma albumin	− 0.07	0.55
Plasma triglycerides	0.08	0.45
Plasma glucose	0.02	0.89
*H*/*L* ratio	− **0.20**	**0.030**
Heterophils	− 0.15	0.11
Lymphocytes	0.08	0.36

Significant correlates (*P* < 0.05) are marked in bold

## Discussion

Our cross-fostering experiment in the common tern showed that nestling immune function was associated with foster colony size. Common tern chicks raised in smaller colonies had stronger skin-swelling response to PHA, which likely reflected lower level of social stress experienced by chicks in smaller breeding aggregations (as indicated by lower nestling *H*/*L* ratios). On the other hand, we found no effect for the original colony size and nutritional condition on nestling PHA response.

Our results corroborated the previous non-experimental findings by Tella et al. (2001), who demonstrated a negative correlation between colony size and PHA response in Magellanic penguin nestlings. Although PHA response of penguin nestlings showed strong positive correlation with body condition (as measured with fledgling mass corrected for structural size), the negative effect of colony size on nestling PHA response remained significant after controlling for variation in condition (Tella et al. 2001). This pattern was interpreted as resulting from an elevated level of social stress experienced by chicks raised in larger colonies. Our study on the common tern provided support for the same mechanism. Although our previous cross-fostering experiments in the common tern colonies showed a negative effect of foster colony size on nutritional state and growth rate of nestlings (Minias et al. 2015), here, we found no relationship between PHA response and condition of chicks. However, we found that nestling PHA response was negatively related to *H*/*L* ratio, which is a proxy of physiological stress. Elevated *H*/*L* ratios occur as a result of white blood cell trafficking, when lymphocytes transmigrate from circulating blood to compartments such as skin, spleen, and lymph nodes, and, simultaneously, there is an influx of heterophils from bone marrow into the blood (Dhabhar et al. 1996; Dhabhar and McEwen 1997). This, so-called, immune-redistribution is probably associated with alterations in animal’s physiology from a state of readiness to cope with a communicable disease (via lymphocytes) to a state of preparation for infection through injury (via heterophils), and it is mediated by corticosteroids (Johnstone et al. 2012). An activation of the hypothalamus–hypophysis–adrenal axis by circulating corticosteroids (in response to environmental or social stress) may also cause a general immunosuppression through inhibition of immune cell function (Braude et al. 1999), thus reducing T-cell-mediated immune response.

We suggest that stress-related mechanisms may well explain a negative effect of foster colony size on PHA-induced skin-swelling response of tern nestlings, as found in this study. It also seems likely that differences in nestling PHA response between colonies of different size were not directly mediated by differences in nesting densities. Although maximum nesting density can positively correlate with colony size in natural conditions (Møller 1982), there were no differences in average nesting densities between our experimental colonies. However, the frequency of interactions between unrelated chicks, as well as between chicks and non-parental adults was likely to be higher in the larger colony because of larger crèche size (max. 120 chicks vs. 30–40 chicks at large and small rafts, respectively). This pattern was due to relatively small area of all the colonies (max. 40 m^2^ for the large raft), which allowed all grown-up chicks to move freely around the entire colonies (see Minias et al. 2015 for details). So far, the effects of environmental or social stress on immune response have been mostly reported for solitary breeding birds. For example, experimental brood enlargement in pied flycatchers *Ficedula hypoleuca* resulted in elevated *H*/*L* ratios and decreased PHA response in nestlings (Ilmonen et al. 2003). In addition, barn swallow *Hirundo rustica* nestlings showed increased corticosterone levels, depressed immune response, and deteriorated body condition in response to either brood enlargement or food deprivation (Saino et al. 2003).

Although our study provided a clear experimental evidence for lower PHA response of nestlings raised in larger colonies, the interpretation of this results must be treated with caution. While PHA response has commonly been used as an index of immunocompetence (Saino et al. 1997; Johnsen et al. 2000; Brommer 2004; Audet et al. 2015), it was also suggested that strong skin-swelling response to PHA may be indicative for an ongoing infection (Biard et al. 2015). In the latter case, stronger PHA response of nestlings raised in smaller colonies would be consistent with higher infection rate. In addition, a recent study on common blackbirds *Turdus merula* demonstrated that interpretation of many immune indices (including PHA response) as evidence for immunocompetence versus ongoing infection might be parasite dependent (Biard et al. 2015). Consequently, PHA response of the same host has been reported to correlate positively with prevalence of some pathogens or parasites and negatively with the others (Biard et al. 2015; Goüy de Bellocq et al. 2007). Here, it is necessary to acknowledge that we failed to collect empirical data on the pathogens or parasites that may be associated with differences in PHA response of common tern nestlings. Although we scanned available blood smears for the presence of hematozoan parasites, we found virtually no evidence for their occurrence in the blood of nestling terns. This result is consistent with the previous screening of adult common terns breeding at the Eastern Coast of the USA, providing support for zero prevalence of hemoparasites in this species (Fiorello et al. 2009). This suggests that hematozoa should not be considered as a major parasite threat for the common tern. Other screenings of pathogen communities in the common tern indicated that the species can harbor various pathogenic bacteria and viruses, including influenza A virus (Olsen et al. 2006), *Campylobacter* and *Yersinia* (Kapperud and Rosef 1983), *Salmonella* (Bogomolni et al. 2008), or *Klebsiella ozaenae* (Rivera et al. 2012). There was also high prevalence of a gastrointestinal protozoan *Cryptosporidium* recorded in common tern nestlings (Rivera et al. 2012). We recommend that future research should focus on testing whether pathogen prevalence and transmission rates vary between waterbird colonies of different size, as well as on linking this variation to more subtle measures of innate and acquired immune response of hosts.

One of our hypotheses predicted that immune function of common tern nestlings may also covary with the size of original colony, assuming that adults choose the colony size based on their own T-cell-mediated immunity and that PHA response is heritable in our population. Specifically, we predicted that adults with lower T-cell-mediated immune response are more likely to settle in smaller colonies to avoid the costs of elevated pathogen transmission rate. However, contrary to our expectation, we did not find any significant effect of original colony size on nestling PHA response in the common tern. First, this may suggest that adult immune function does not affect their settlement decisions in the common tern. As far as we are aware, the hypothesis of immunity-based sorting of individuals among colonies of different size has not been directly tested in birds and it merits further research. However, it is also possible that PHA response in our study population does not have a strong genetic component. Although skin-swelling response to PHA was shown to be heritable in poultry (Cheng and Lamont 1988), studies on wildbird species provided mixed evidence. Full-sib comparisons in cross-fostering experiments indicated significant heritability of PHA response in some avian species (Brinkhoff et al. 1999; Ardia 2005; Kim et al. 2013), but not in the others (Christe et al. 2000; Kipimaa et al. 2005). Implementation of animal model approach that separates additive genetic effects from dominance variance and early environmental (maternal) effects (Kruuk 2004) indicated very low narrow-sense heritability of PHA response in several passerine species (Pitala et al. 2007; Sakaluk et al. 2014). In addition, it is acknowledged that gene expression may vary across environments (genotype-by-environment interaction), resulting in large between-population variation in heritability of immune function (Ardia and Rice 2006). Thus, based on the previous research in wild-living birds, it seems unsafe to a priori assume heritability of nestling PHA response in our study population.

In conclusion, our cross-fostering experiment in the common tern provided the first experimental evidence for the negative effect of colony size on nestling immune function, which was most likely mediated by inter-colony variation in the level of social stress. Nestling PHA response is generally acknowledged to determine long-term survival prospects and it predicted local recruitment in a number of avian species (Cichoń and Dubiec 2005; Moreno et al. 2005; López-Rull et al. 2011). Thus, depression of nestling immune response via social stress may constitute a strong selective pressure against large colony size in the common tern, and possibly in other colonial species. We recommend that this largely overlooked cost of sociality should be considered in the further studies on the evolution and ecology of avian coloniality.

## Electronic supplementary material

Below is the link to the electronic supplementary material.

Supplementary file1 (XLSX 29 kb)
